# Dynamics of hospitalizations and staffing of Ukraine’s mental health services during the Russian invasion

**DOI:** 10.1186/s13033-023-00589-4

**Published:** 2023-06-24

**Authors:** Irina Pinchuk, Ryunosuke Goto, Oleksiy Kolodezhny, Nataliia Pimenova, Norbert Skokauskas

**Affiliations:** 1 Institute of Psychiatry, Taras Shevchenko National University of Kyiv, Kyiv, Ukraine; 2 Department of Pediatrics, The University of Tokyo Hospital, Tokyo, Japan; 3 Regional Centre for Children and Youth Mental Health and Child Welfare - Central Norway, IPH, Norwegian University of Science and Technology, RKBU Midt-Norge, NTNU, Postboks 8905 MTFS, NO-7491, Trondheim, Norway; 4 Child and Adolescent Psychiatry Section, World Psychiatric Association (WPA), Geneva, Switzerland

**Keywords:** Humanitarian health, Global health, Global mental health, Health services research, Health policy, Psychiatry

## Abstract

**Background:**

Since February 2022, the people of Ukraine have experienced devastating losses due to the Russian invasion, increasing the demand for mental healthcare across the nation. Using longitudinal data on mental health facilities across the nation up to summer 2022, we aimed to provide an updated picture of Ukrainian mental health services during the 2022 Russian invasion.

**Methods:**

We conducted a nationwide longitudinal study on Ukrainian inpatient mental health facilities during the Russian invasion since February 2022. We obtained responses from the heads of 30 inpatient mental health facilities, which represent 49.2% of all psychiatric hospitals in Ukraine. Information on hospitalizations and the number, displacement, and injuries of staff in April and July-September 2022 was obtained from each facility.

**Results:**

Facilities across Ukraine reported similar staff shortages in both April and August-September 2022, despite an increase in the number of hospitalizations in July 2022 and a similar percentage of hospitalizations related to war trauma (11.6% in July vs. 10.2% in April, Wilcoxon signed-rank test P = 0.10). Hospitalizations related to war trauma became more dispersed across the nation in July 2022, likely reflecting the return of internally and externally displaced persons to their original locations.

**Conclusions:**

The mental health needs and services changed drastically in the first half-year of the Russian invasion of Ukraine, with those in need more dispersed across the country over time. International aid may need to be scaled up to stably provide mental healthcare, given the displacement of the mental healthcare workforce.

## Introduction

In a previous study, we found that the mental health service structure in Ukraine was severely damaged early in the Russian invasion in 2022, with staff shortages despite a significant number of hospitalizations related to war trauma [1]. Resource shortages, especially human resource shortages, were especially pronounced in areas of active hostilities and in eastern regions of Ukraine, which at the time were directly occupied by the Russian forces.

All of these damages were on top of the already vulnerable Ukrainian mental healthcare system – even prior to the war, Ukraine had significant mental health needs and limited resources to address them. For example, whereas 12.4% of adults in Ukraine had symptoms consistent with a diagnosis of depression, only 3.2% of individuals with depression received treatment [2]. Earlier, we found that inpatient mental health services in Ukraine (which remains the backbone of the nation’s mental healthcare, with recent efforts to shift to a community-centered approach that have been slowed down by the COVID-19 pandemic) [3] were heavily damaged in areas directly attacked by the Russian forces [1], and Ukraine is arguably in need of a stronger mental healthcare system amidst the war. Though previous studies suggest that mental health care needs increase drastically during war and that the resilience of mental healthcare workforce needs to be strengthened, they seem only to give a vague picture as to what specifically the needs of mental health facilities are [4, 5]. Thus, updated evidence on mental health services in Ukraine, and mental health services in war situations in general, is necessary.

Many problems arise with chronic exposure to war, ranging from basic resource shortages to health issues due to war trauma [1]. For instance, war disrupts care for pre-existing health problems [6, 7], which could result in substantial morbidity and mortality in the civilian population. The burden on the mental health of war-exposed populations is especially significant, with risk of numerous psychiatric sequelae stemming from trauma exposure [8]. Such consequences start in early childhood, with evidence for delays in socioemotional development in children as young as three or four years old [9]. Among adults and non-civilian populations exposed to war, psychiatric disorders such as post-traumatic stress disorder (PTSD) are highly prevalent: in a 2016 study using a representative sample of more than 2,000 internally-displaced adults in Ukraine, nearly 30% were found to have PTSD [10], despite that in 2016 the intensity of the war in Ukraine was lower than it is now.

With multiple calls for action to address Ukraine’s mental health needs [11–13], information on the viability of inpatient mental health services in Ukraine, which remains the backbone of the nation’s psychiatric services, needs to be updated. Using longitudinal data on mental health facilities across the nation up to September 2022, we aimed to provide an updated picture of Ukrainian mental health services during the 2022 Russian invasion.

## Method

To assess the state of mental health services in Ukraine as of September 2022, we conducted two waves of online surveys to heads of inpatient mental health facilities in Ukraine. Ukraine has 27 (including Crimea and Sevastopol) regions, each of which has one main psychiatric hospital, and many regions have several other psychiatric hospitals in addition to the main hospital. In each wave, we contacted the heads of each of the main psychiatric hospitals in 25 (all regions in Ukraine excluding Crimea and Sevastopol) regions of Ukraine via online messaging, e-mails, and calls. In addition to answering about their own hospitals, the directors of the main hospitals were asked to distribute the questionnaires to other psychiatric hospitals in the region. Of 61 psychiatric hospitals in Ukraine, we were able to recruit 37 in the first wave and 34 in the second wave. In the first wave, data were collected from May 2nd to June 2nd, 2022, and in the second wave, data were collected from August 16th to September 3rd, 2022. The director of each facility provided consent to participate in the study by answering the questionnaire. The study was approved by the ethics committee of Taras Shevchenko National University of Kyiv’s Institute of Psychiatry (No. 6/16.08.2022). Only facilities that participated in both waves were included in the study.

The directors of facilities provided information on the mental health services at their facilities before the start of the 2022 invasion (January 2022; participants were asked to retrospectively provide information in the first wave), in April 2022 (first wave; data were collected from May 2nd to June 2nd, 2022), and in July-September 2022 (second wave; data were collected from August 16th to September 3rd, 2022). They were asked about the number of inpatient beds, total hospitalizations, hospitalizations related to war trauma (referring to any mental illnesses secondary to exposure to war), number of staff (psychiatrists, nurses, junior nurses, psychologists, and social workers), number of injured workers, and number of displaced workers. In the first survey, respondents were asked to provide information about April 2022, and in the follow-up survey, they were asked provide information on hospitalizations in July 2022 and information on the number, injuries, and displacement of staff at the time of the study (August-September 2022). Comparisons of data before and during the war as well as across waves were conducted using Wilcoxon signed-rank tests. To compute percent changes in hospitalizations and percentages of hospitalizations related to war trauma across facilities in the first and second waves, we weighted these proportions by the number of hospitalizations per facility in January 2022. Similarly, percentages of injured workers and displaced workers were weighted by the number of total medical workers per facility in January 2022.

To visualize the changes in these outcomes between the first wave and the second wave, we created Sankey diagrams for the percentages of hospitalizations related to war trauma and percentages of displaced workers (out of total medical workers). We then created proportional symbol maps of percentages of hospitalizations related to war trauma for each of the two waves, percentages of displaced workers (out of total medical workers) for each of the two waves, and percentages of new employees out of total medical workers in August-September 2022. Data from the two waves were overlaid to visualize the changes over time, and sites of territorial occupation by Russia were also plotted on the maps. The data on territorial occupation were based on manually curated military event reports indicating whether the administrative center or other major city of each district are/were occupied by the Russian forces by August 16th, 2022 [14]. The data used for the Sankey diagrams and maps were aggregated by region to protect the anonymity of the facilities. In our analyses on hospitalizations related to war trauma, we excluded facilities that did not have any hospitalizations prior to the start of the invasion. All analyses were conducted using R version 4.1.1.

## Results

**Fig. 1:**
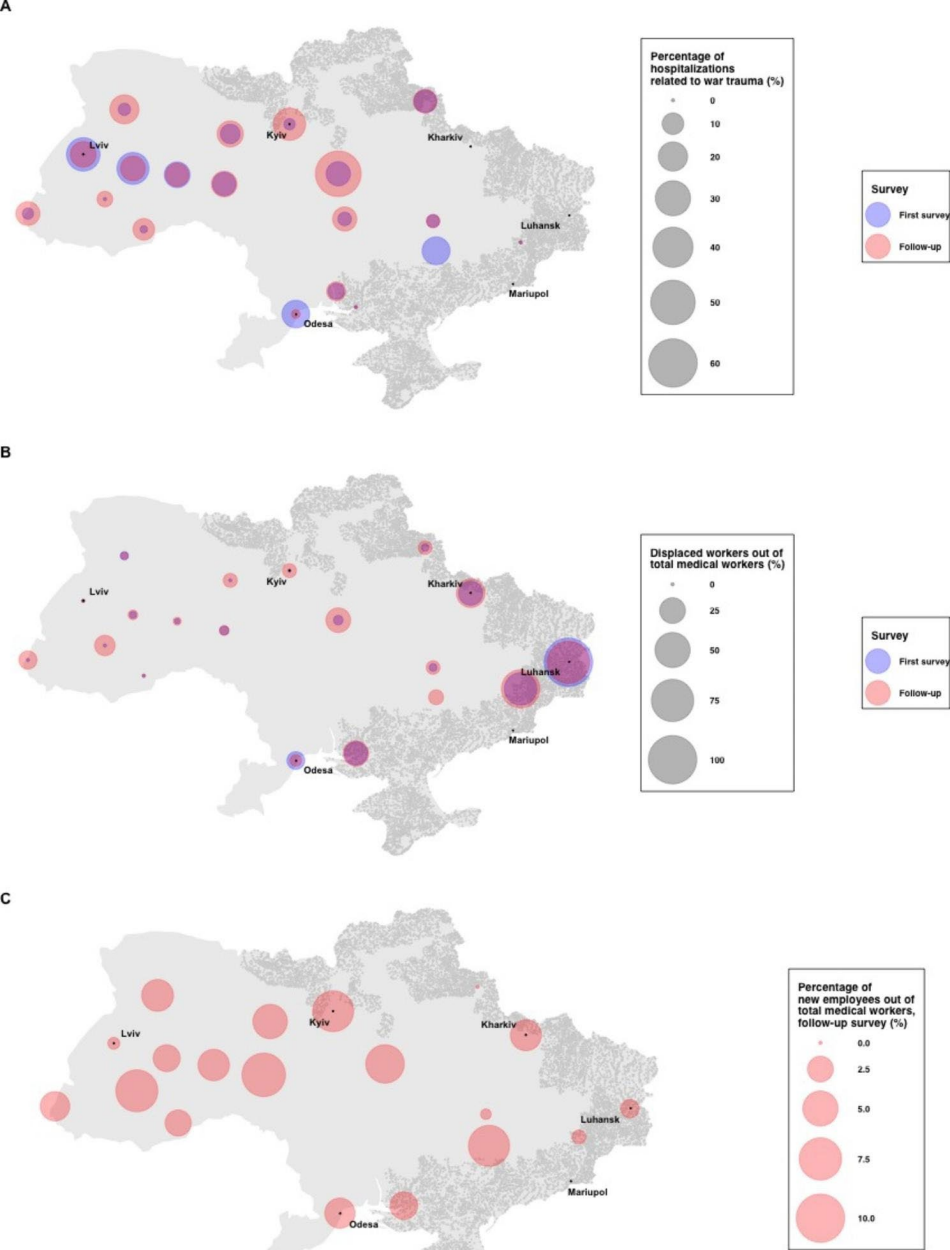
Proportional symbol maps of changes in mental health services in Ukraine during the 2022 Russian invasion. Proportional symbol maps were created for (**A**) percentages of hospitalizations related to war trauma in each the two waves (%), (**B**) percentages displaced workers out of total medical workers in each of the two waves (%), and (**C**) percentages of new employees out of total medical workers in the follow-up survey (%). For the follow-up survey, facilities reported information on hospitalizations in July 2022 and information on the number and displacement of staff at the time of the study (August-September 2022). Each circle represents the percentages aggregated by region. Shaded areas (in grey) represent regions that are or have been under Russian occupation as of August 16th, 2022. All hospitals with more hospitalizations in the first survey or the follow-up survey than January 2022 are shown to have no reductions in hospitalizations (0%). Note that percentages of displaced workers out of total medical workers could exceed 100% as some workers may not have been medical workers. Statistics for regions with unavailable data are not shown

**Fig. 2:**
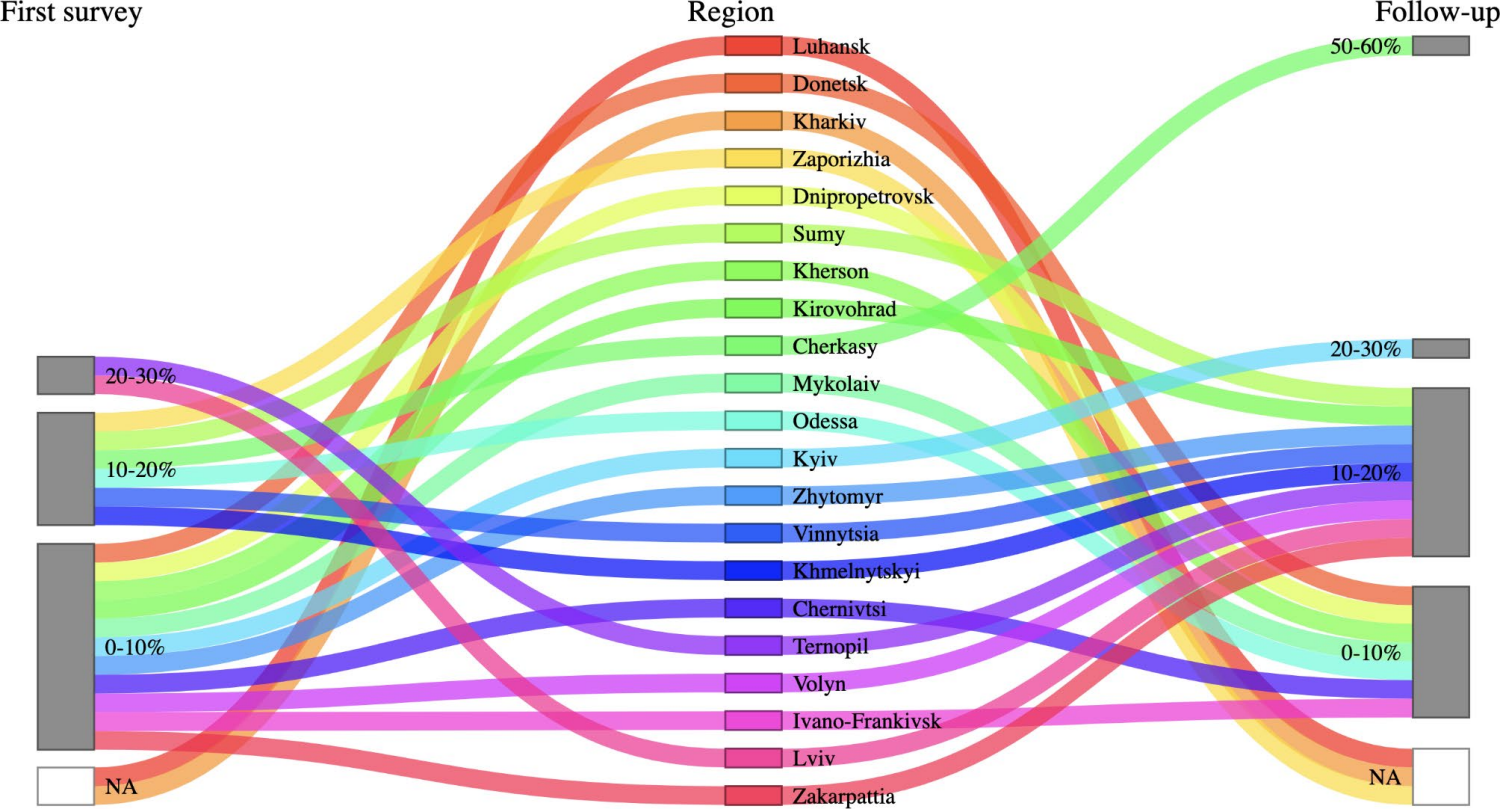
Sankey plot of percentages of hospitalizations related to war trauma, by region. Regions are aligned from east to west. Facilities reported hospitalizations in April and July 2022 for the first and follow-up surveys, respectively

30 facilities that participated in both the first and the follow-up study, which represent 49.2% of all psychiatric hospitals in Ukraine, were included in the analyses. The average number of inpatient beds per facility was 380.9 beds. There were fewer hospitalizations in April 2022 compared to before the war (January 2022) (333.7 vs. 432.2 per month, Wilcoxon signed-rank test P = 0.002), but hospitalizations rose in July 2022 compared to April 2022 (540.9 vs. 333.7 per month, Wilcoxon signed-rank test P < 0.001, Table 1). Across facilities, 11.6% of hospitalizations in July 2022 were related to war trauma, comparable to the 10.2% of hospitalizations in April 2022 (Wilcoxon signed-rank test P = 0.10, Table 1). In April 2022, facilities with the largest percentages of hospitalizations related to war trauma were located in regions with internally displaced persons (Fig. 1A). Hospitalizations related to war trauma became more dispersed across the nation in July 2022 (Fig. 1A). Notably, in April 2022, there were more hospitalizations related to war trauma in Lviv and Ternopil regions, whereas in July 2022, a significant proportion of hospitalizations related to war trauma was noted in the Cherkasy region, as well as in Kyiv (Fig. 2).

**Table 1:** Basic characteristics

Facility characteristics (N = 30)	Baseline, January 2022	First survey, April 2022	Follow-up survey, July-September 2022
Number of inpatient beds	380.9 (282.5)		
Hospitalizations	432.2 (276.8)	333.7 (291.3)	540.9 (744.0)
Reduction in hospitalizations compared to baseline (%)	-	22.8	-37.8%
Percent of hospitalizations related to war trauma (%)	-	10.2%	11.6%
Number of psychiatrists	34.9 (27.4)	31.7 (24.8)	29.6 (24.1)
Number of nurses	142.6 (119.5)	129.6 (117.6)	122.9 (101.4)
Number of junior nurses	153.4 (118.1)	138.0 (112.4)	139.5 (113.9)
Number of psychologists	12.8 (36.0)	5.7 (5.3)	6.1 (6.0)
Number of social workers	1.8 (2.1)	1.6 (1.7)	1.4 (1.6)
Injured workers out of total medical workers (%)	-	0.5%	3.5%
Displaced workers out of total medical workers (%)	-	10.1%	12.1%

Facility-level characteristics were expressed as mean (standard deviation) or as weighted percentages. A negative percentage reduction in hospitalization represents an increase in hospitalizations. Baseline data was collected retrospectively during the first wave. For the follow-up survey, facilities reported information on hospitalizations in July 2022 and information on the number, injuries, and displacement of staff at the time of the study (August-September 2022)

In August-September 2022, there were fewer psychiatrists than prior to the start of the 2022 invasion (29.6 vs. 34.9 per facility, Wilcoxon signed-rank test P = 0.04), but not in April 2022 compared to pre-war (31.7 vs. 34.9 per facility, P = 0.46). There were fewer nurses (122.9 vs. 142.6 per facility, P = 0.001), junior nurses (139.5 vs. 153.4 per facility, P = 0.004), and psychologists (6.1 vs. 12.8 per facility, P = 0.07) in August to September 2022 compared to before the war, similar to comparisons between April 2022 and before the war (P = 0.02, 0.05, and 0.03 for nurses, junior nurses, and psychologists, respectively). We did not find strong evidence of a smaller number of social workers in both August to September 2022 (1.4 vs. 1.8 per facility, P = 0.10) and April 2022 (1.6 vs. 1.8 per facility, P = 0.17) compared to before the war (Table 1).

**Fig. 3:**
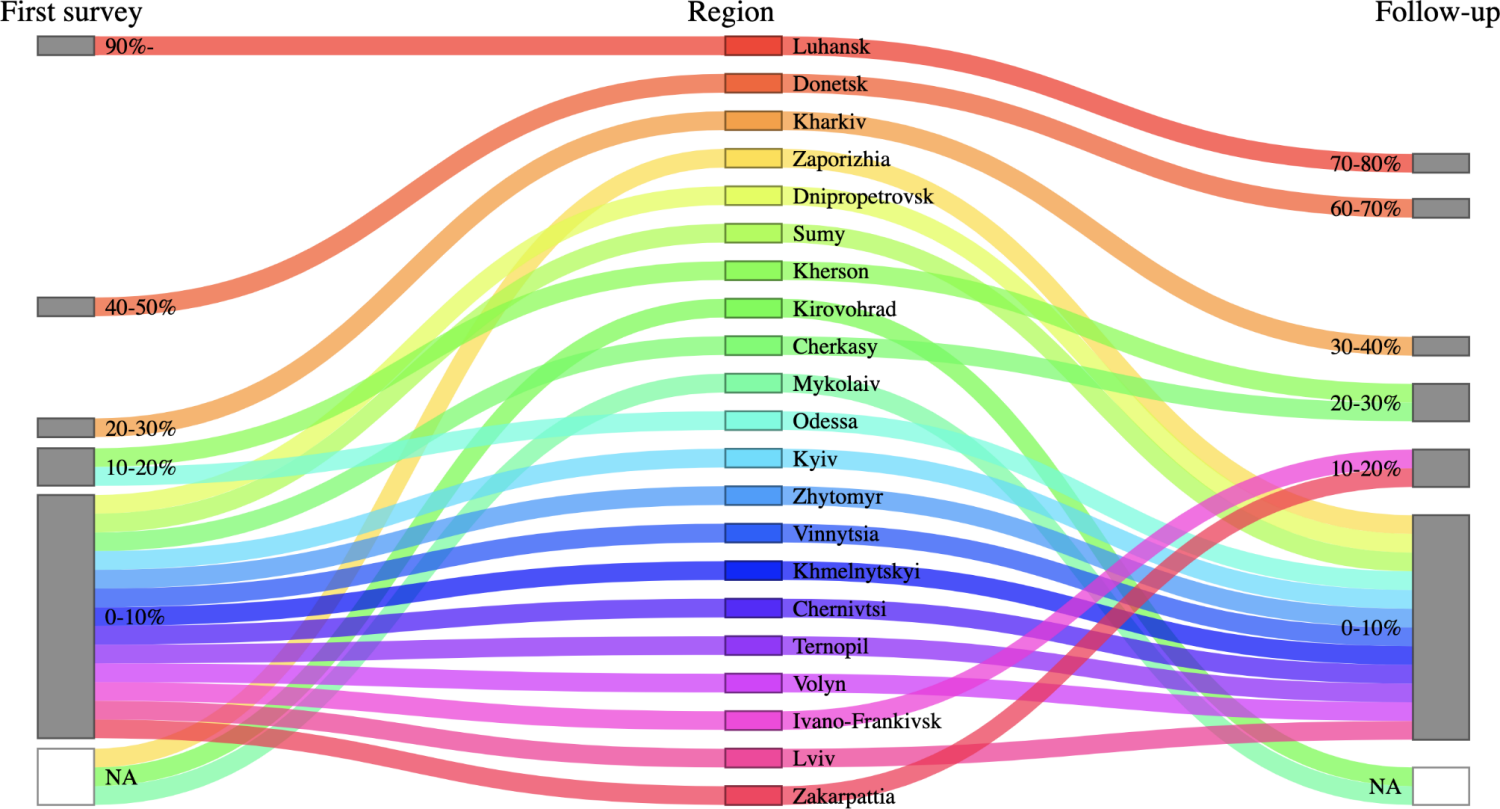
Sankey plot of percentages of displaced workers out of total medical workers, by region. Regions are aligned from east to west. Facilities reported numbers of displaced workers in April and August to September 2022 for the first and follow-up surveys, respectively

Across facilities, in August-September 2022, 12.1% of the total medical workers were displaced and 3.5% were injured. Although in April 2022, facilities with displaced workers were largely concentrated around regions directly occupied by the Russian forces and in areas of active hostilities, such facilities were more dispersed across the nation in August to September 2022 (Fig. 1B). Notably, Donetsk, Kharkov, Kherson, and Cherkasy regions had more displacements in August-September compared to April 2022. Some patients and staff were evacuated from the Donetsk and Kharkiv regions to the western regions of the country (Fig. 3), as hospitals in these regions, along with Mariupol, reported direct damages from the war. Despite this, facilities across Ukraine reported new staff joining their workforce in August-September 2022 (Fig. 1C).

## Discussion

In general, hospitalizations across Ukraine increased from April to July 2022, and hospitalizations related to war trauma remained unchanged during this period. Mental health facilities across Ukraine reported displaced workers in both surveys, and despite new employees joining the workforce in August-September 2022, facilities had fewer healthcare workers compared to before the start of the war.

More specifically, in April 2022, there were more hospitalizations related to war trauma in Lviv, Ternopil, Khmelnytsky and Vinnytsia regions, as well as in Odessa and Zaporizhia regions, which were part of the evacuation routes of Ukrainians. In July 2022, the situation changed. A significant proportion of hospitalizations were related to war trauma in the Cherkasy region as well as in Kyiv (most likely due to the return of the local population and an increase in the number of temporarily displaced people from eastern Ukraine who experienced war trauma), Kirovohrad, Zhytomyr, Volyn, and Zakarpattia regions. This is a large change in comparison to our first study [15], and our findings reflect the migration of the population in Ukraine in different periods of the war, depending on the activity of hostilities in different parts of the country. It is interesting to note that a significant decrease in hospitalizations related to war trauma occurred in the Donetsk region; hospitals in the region suffered direct damages from the war.

Our study revealed a changing landscape of mental health needs and services during the 2022 Russian invasion of Ukraine, as reflected by the changes in the distribution of hospitalizations related to war trauma and in the number of staff. Importantly, policymakers and organizations providing international aid need to be aware that the mental health needs of war-affected populations may change dynamically, as was the case in the Russian invasion of Ukraine. For instance, international mental healthcare aid should be allocated not only to regions most drastically affected by war (in the case of Ukraine, the eastern regions), but other parts of the country as well, since people affected by war trauma become more dispersed over time. Importantly, the increased mental health needs during the invasion were amidst the shrinkage of the healthcare workforce due to displacement, trauma, injury, and, sometimes, death of healthcare workers, as reflected by the results of the present study. These findings are alarming, as this would mean that meeting the healthcare needs of the Ukrainian population is very challenging at status quo, especially its mental health workforce. International aid may need to be scaled up to meet the increased demand for mental healthcare.

Our study has limitations. As with our previous study [1], there may have been measurement bias due to the self-reported nature of the questionnaires, and the fact that some facilities did not participate in our study may limit the representativity of our sample. However, to our knowledge, our studies provide the most up-to-date information on mental health service availability in Ukraine during the ongoing Russian invasion. As the data collected in this study are from the heads of psychiatric hospitals and not from the people affected from the war themselves, the actual mental health needs of the civilian and combatant population ought to be assessed through direct evaluations.

## Data Availability

Data used for this study are not publicly available.

## List of abbreviations

PTSD: Post-traumatic stress disorder
