# Correction: Dynamics of hospitalizations and staffing of Ukraine’s mental health services during the Russian invasion

**DOI:** 10.1186/s13033-024-00660-8

**Published:** 2025-01-07

**Authors:** Irina Pinchuk, Ryunosuke Goto, Oleksiy Kolodezhny, Nataliia Pimenova, Norbert Skokauskas

**Affiliations:** 1 Institute of Psychiatry, Taras Shevchenko National University of Kyiv, Kyiv, Ukraine; 2 Department of Pediatrics, The University of Tokyo Hospital, Tokyo, Japan; 3 Regional Centre for Children and Youth Mental Health and Child Welfare - Central Norway, IPH, Norwegian University of Science and Technology, RKBU Midt-Norge, NTNU, Postboks 8905 MTFS, Trondheim, NO-7491 Norway; 4 Child and Adolescent Psychiatry Section, World Psychiatric Association (WPA), Geneva, Switzerland

**International Journal of Mental Health Systems (2023) 17:20**

10.1186/s13033-023-00589-4

Following publication of the original article, the authors would like to correct mean and SD of hospitalizations reported in July 2022, which also impacted the comparisons to baseline and first wave survey data.

The sentence currently reads:

There were fewer hospitalizations in April 2022 compared to before the war (January 2022) (333.7 vs. 432.2 per month, Wilcoxon signed-rank test *P* = 0.002), but hospitalizations rose in July 2022 compared to April 2022 (540.9 vs. 333.7 per month, Wilcoxon signed-rank test *P* < 0.001, Table 1). Across facilities, 11.6% of hospitalizations in July 2022 were related to war trauma, comparable to the 10.2% of hospitalizations in April 2022 (Wilcoxon signed-rank test *P* = 0.10, Table 1).

The sentence should read:

There were fewer hospitalizations in April 2022 compared to before the war (January 2022) (333.7 vs. 432.2 per month, Wilcoxon signed-rank test *P* = 0.002), but hospitalizations rose in July 2022 compared to April 2022 (**360.9** vs. 333.7 per month, Wilcoxon signed-rank test ***p*** **= 0.002**, Table 1).

The authors identified an error in Table 1.

In Table 1: Raw ‘Hospitalizations’ in column ‘Follow-up survey, July-September 2022’: **360.9 (316.7).** Raw ‘Reduction in hospitalizations compared to baseline (%)’ in column ‘Follow-up survey, July-September 2022’: **16.5%.**

Incorrect Table 1.

**Figure:**
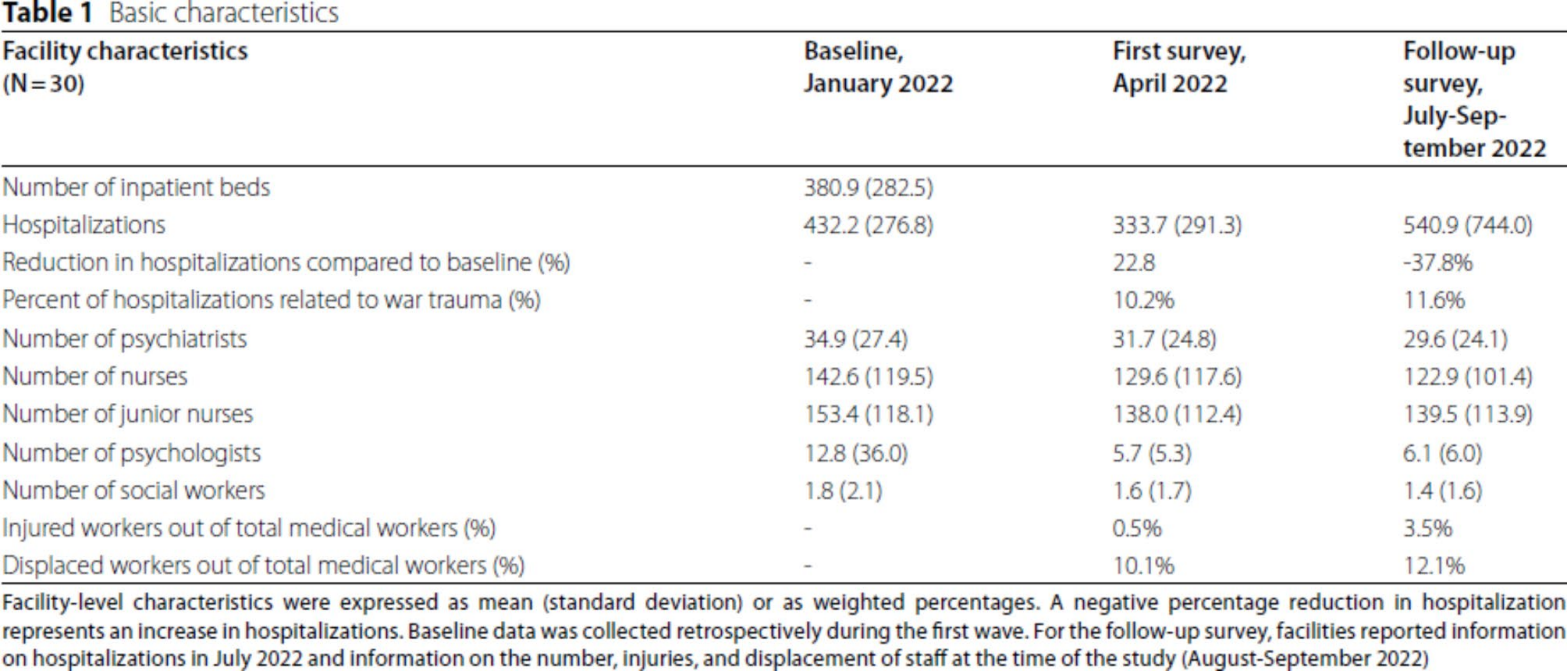

Corrected Table 1.

**Table 1:** Basic characteristics

Facility characteristics (*N* = 30)	Baseline, January 2022	First survey, April 2022	Follow-up survey, July-September 2022
Number of inpatient beds	380.9 (282.5)		
Hospitalizations	432.2 (276.8)	333.7 (291.3)	360.9 (316.7)
Reduction in hospitalizations compared to baseline (%)	-	22.8%	16.5%
Percent of hospitalizations related to war trauma (%)	-	10.2%	11.6%
Number of psychiatrists	34.9 (27.4)	31.7 (24.8)	29.6 (24.1)
Number of nurses	142.6 (119.5)	129.6 (117.6)	122.9 (101.4)
Number of junior nurses	153.4 (118.1)	138.0 (112.4)	139.5 (113.9)
Number of psychologists	12.8 (36.0)	5.7 (5.3)	6.1 (6.0)
Number of social workers	1.8 (2.1)	1.6 (1.7)	1.4 (1.6)
Injured workers out of total medical workers (%)	-	0.5%	3.5%
Displaced workers out of total medical workers (%)	-	10.1%	12.1%

Facility-level characteristics were expressed as mean (standard deviation) or as weighted percentages. A negative percentage reduction in hospitalization represents an increase in hospitalizations. Baseline data was collected retrospectively during the first wave. For the follow-up survey, facilities reported information on hospitalizations in July 2022 and information on the number, injuries, and displacement of staff at the time of the study (August-September 2022).

Incorrect Fig. 1.

**Figure:**
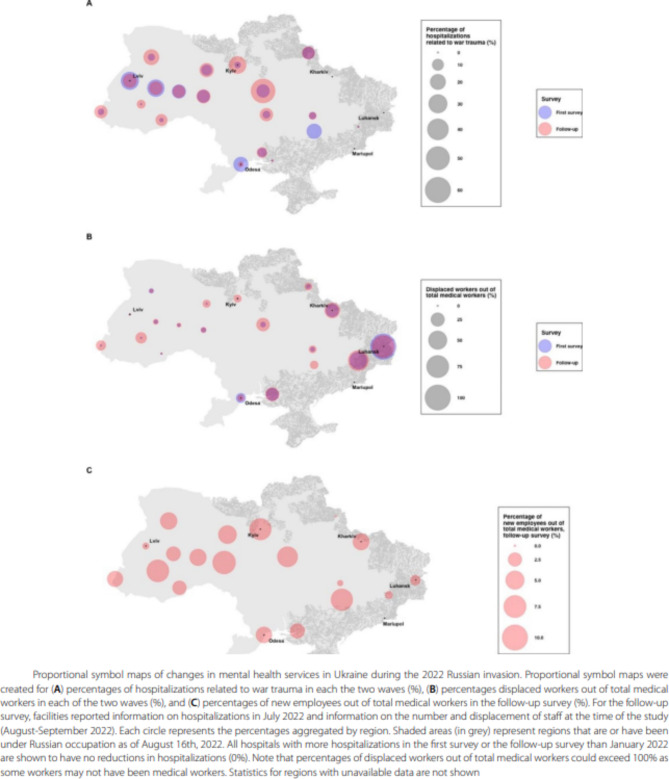

Corrected Fig. 1. 

**Fig. 1:**
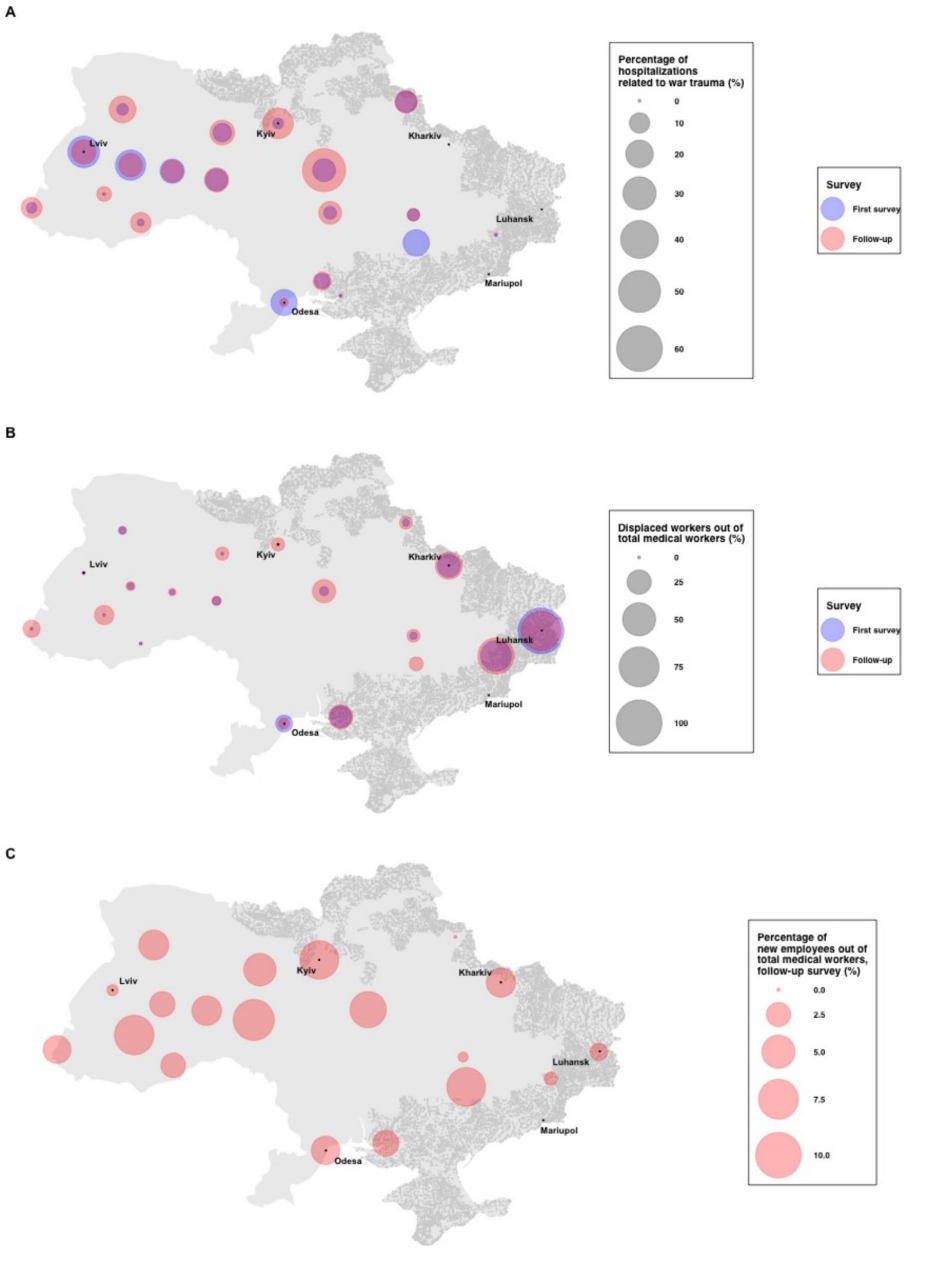
Proportional symbol maps of changes in mental health services in Ukraine during the 2022 Russian invasion. Proportional symbol maps were created for **(A)** percentages of hospitalizations related to war trauma in each the two waves (%), **(B)** percentages displaced workers out of total medical workers in each of the two waves (%), and **(C)** percentages of new employees out of total medical workers in the follow-up survey (%). For the follow-up survey, facilities reported information on hospitalizations in July 2022 and information on the number and displacement of staff at the time of the study (August-September 2022). Each circle represents the percentages aggregated by region. Shaded areas (in grey) represent regions that are or have been under Russian occupation as of August 16th, 2022. All hospitals with more hospitalizations in the first survey or the follow-up survey than January 2022 are shown to have no reductions in hospitalizations (0%). Note that percentages of displaced workers out of total medical workers could exceed 100% as some workers may not have been medical workers. Statistics for regions with unavailable data are not shown.

Incorrect Fig. 2.

**Figure:**
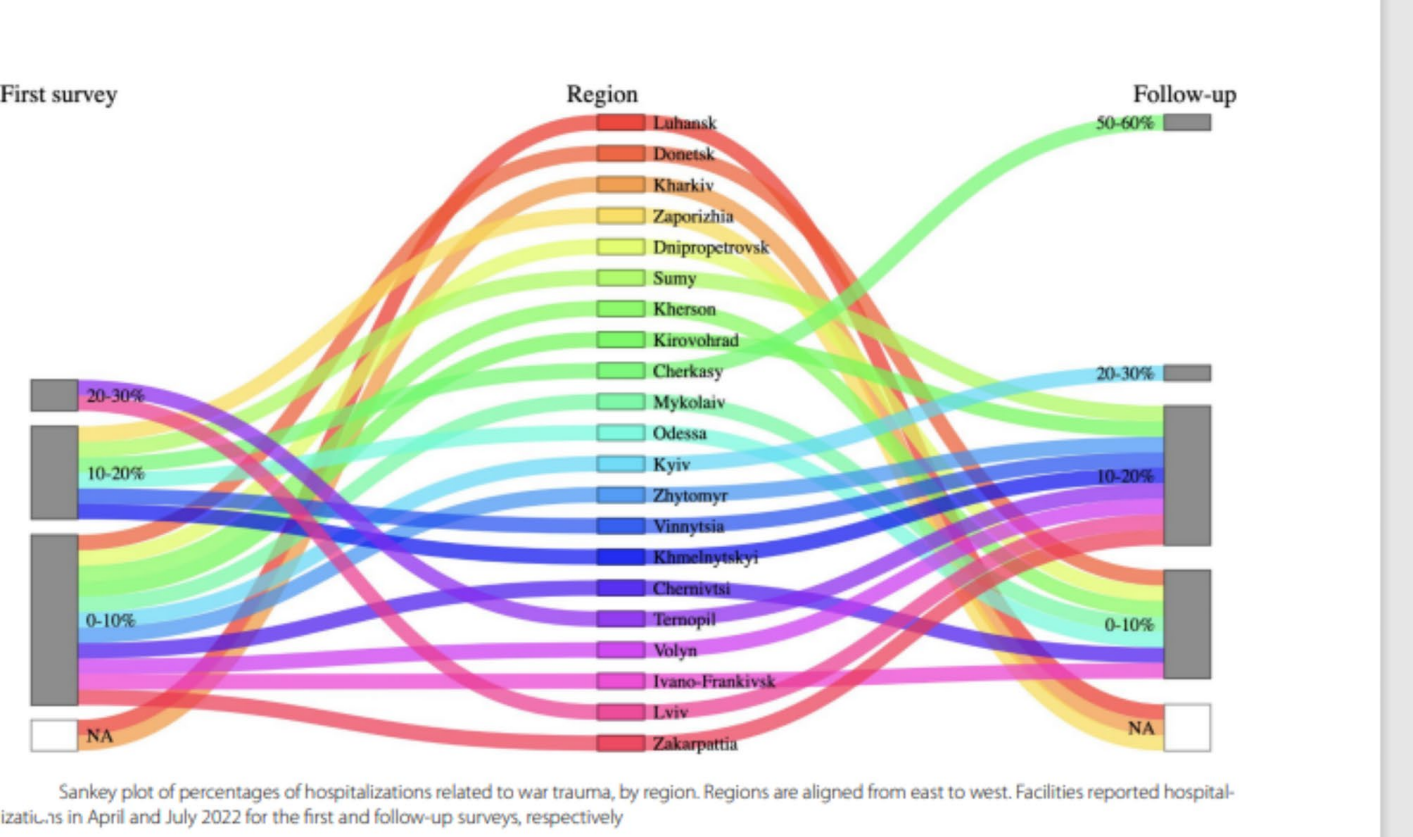

Corrected Fig. 2:

**Fig. 2:**
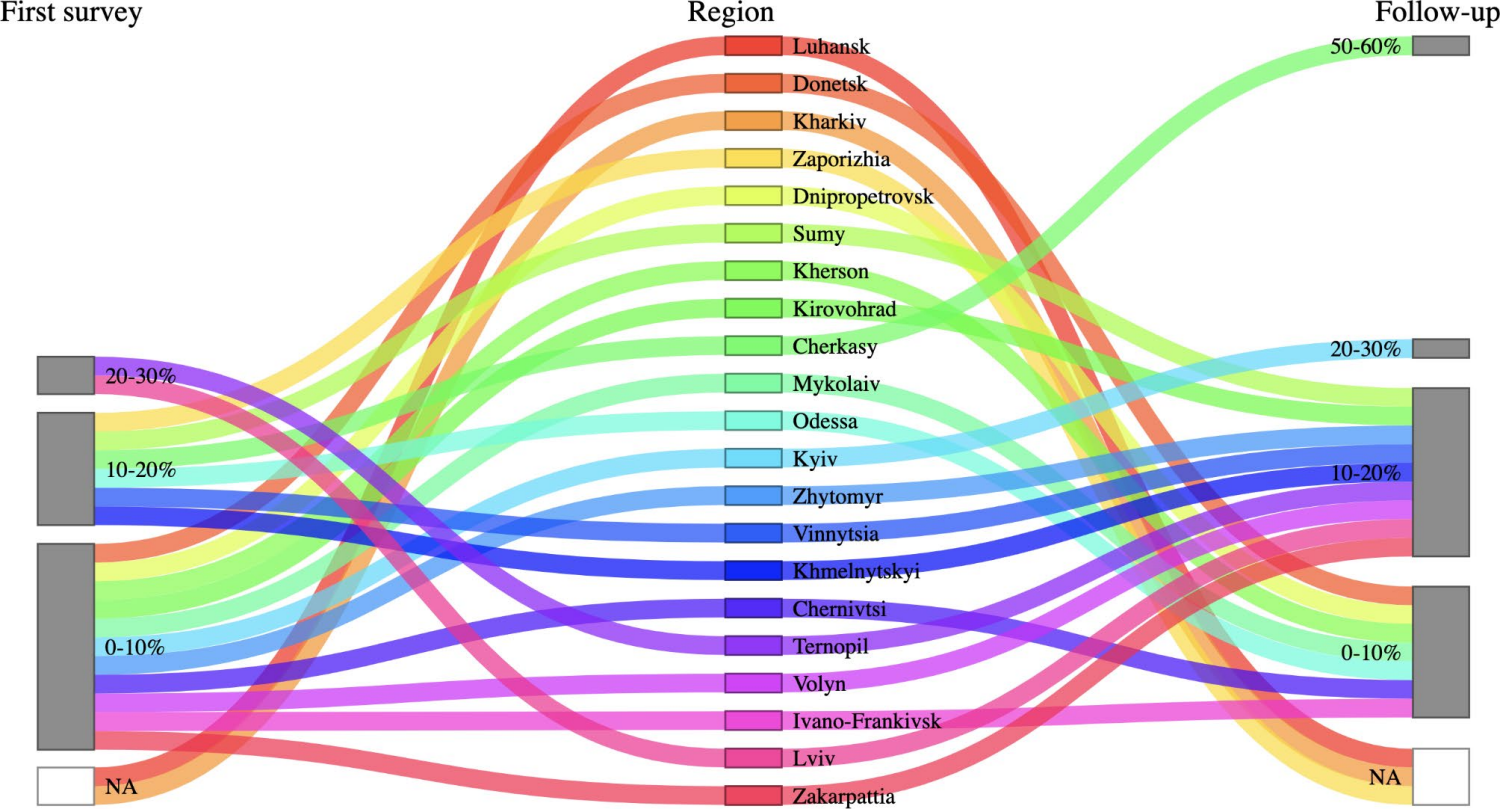
Sankey plot of percentages of hospitalizations related to war trauma, by region. Regions are aligned from east to west. Facilities reported hospitalizations in April and July 2022 for the first and follow-up surveys, respectively

Incorrect Fig. 3:

**Figure:**
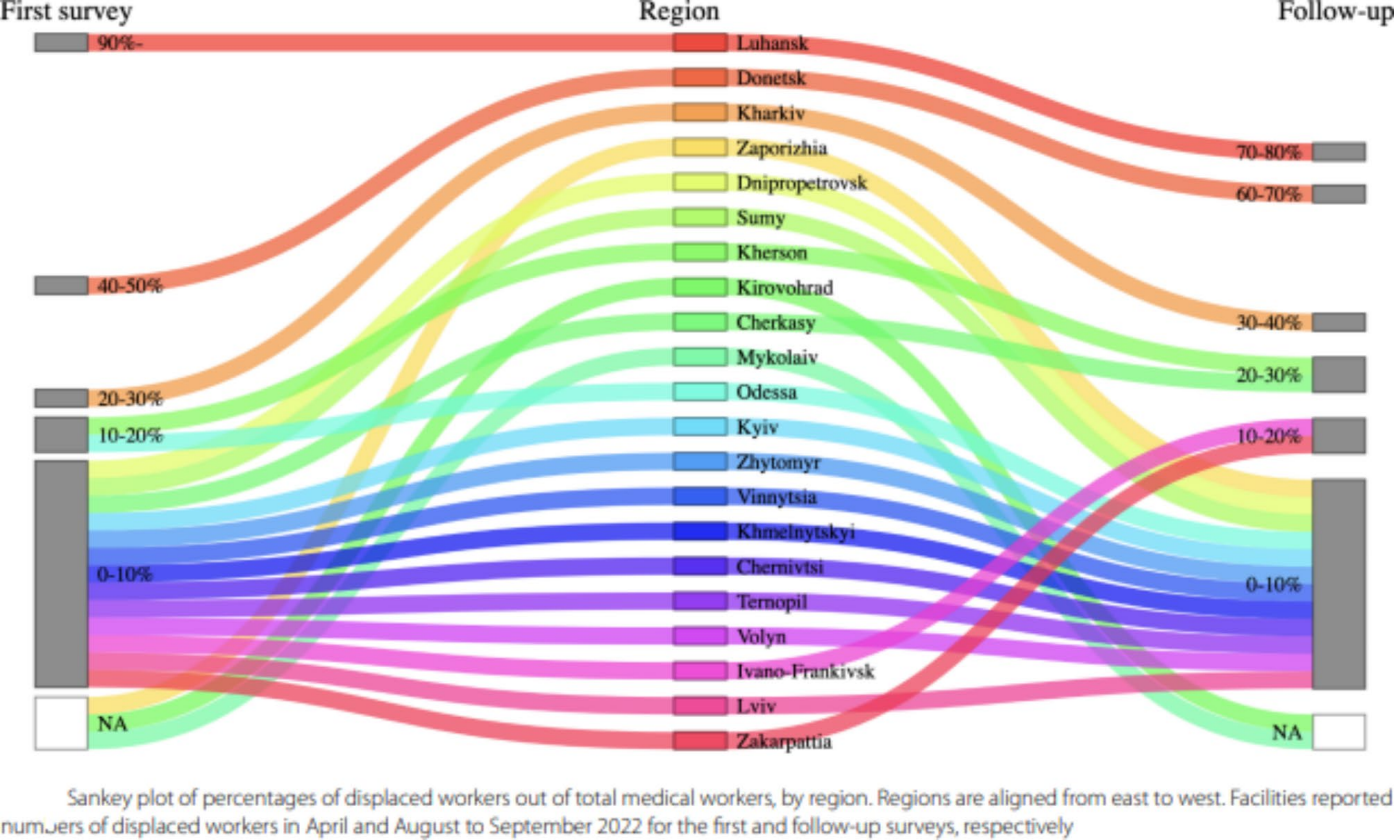

Corrected Fig. 3:

**Fig. 3:**
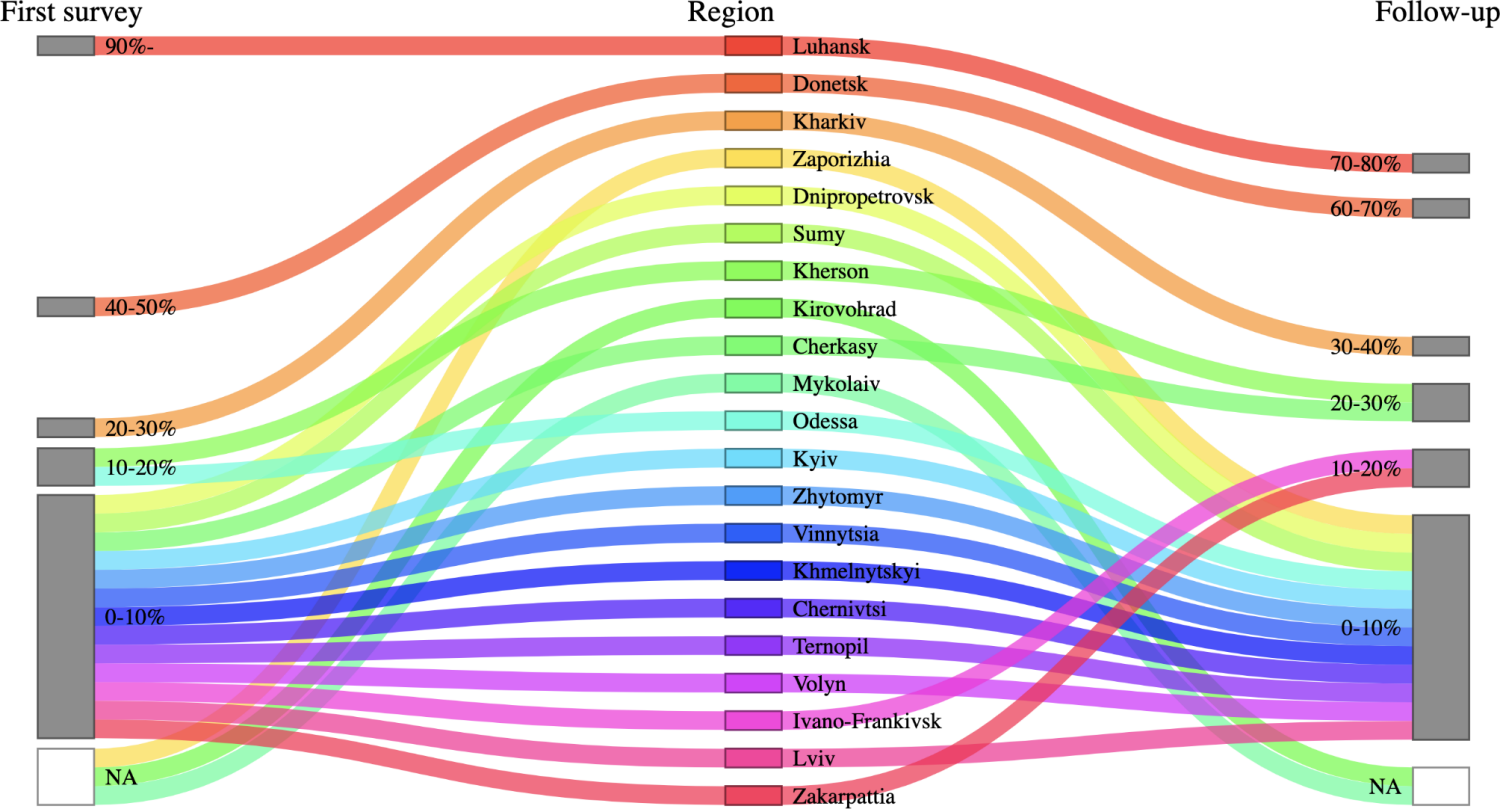
Sankey plot of percentages of displaced workers out of total medical workers, by region. Regions are aligned from east to west. Facilities reported numbers of displaced workers in April and August to September 2022 for the first and follow-up surveys, respectively

The authors note that this error resulted from inaccuracies in data entry and an inability to verify data from certain hospitals located near occupied territories at the time of the study.

To ensure the accuracy and integrity of the published record, the original article has been corrected.

